# Changes in Human Electroencephalographic Activity in Response to *Agastache rugosa* Essential Oil Exposure

**DOI:** 10.3390/bs12070238

**Published:** 2022-07-15

**Authors:** Minji Hong, Hyejeong Jang, Sela Bo, Minju Kim, Ponnuvel Deepa, Jiyea Park, Kandhasamy Sowndhararajan, Songmun Kim

**Affiliations:** 1 School of Natural Resources and Environmental Science, Kangwon National University, Chuncheon 24341, Korea; alswl0356@kangwon.ac.kr (M.H.); gpwjd6576@naver.com (H.J.); bosela80@gmail.com (S.B.); scent@kangwon.ac.kr (M.K.); taanishadeepa@gmail.com (P.D.); 2 Bigsome Inc., 501 Jinju-daero, Jinju 52828, Korea; jiyea35@hanmail.net; 3 Department of Botany, Kongunadu Arts and Science College, Coimbatore 641029, India; sowndhar1982@gmail.com

**Keywords:** *Agastache rugosa*, Korean mint, essential oil, estragole, electroencephalographic activity

## Abstract

*Agastache rugosa* (Korean mint) is an important medicinal and aromatic plant and its aerial parts have a pleasant fragrance. *A. rugosa* leaves are used as an ingredient in salads and soups for enhancing the aroma and taste of foods in Korea. However, there is no report on the influence of the aroma of *A. rugosa* on human psychophysiological activity. Therefore, the present study aimed to investigate the effect of exposure to the essential oil of Korean *A. rugosa* on human electroencephalographic (EEG) activity. The essential oil of *A. rugosa* was isolated using steam distillation extraction and its composition was determined by gas chromatography and mass spectrometry (GC–MS) analysis. In the EEG study, 38 healthy volunteers (19 men and 19 women) participated. The EEG readings were analyzed for 25 EEG indices from 29 electrodes placed on the scalp according to the international 10–20 system. The major component in the essential oil of *A. rugosa* was estragole (89.49%) followed by D-limonene (3.40%), menthone (1.80%), and pulegone (1.86%). In the EEG study, significant decreases in absolute theta (AT) and relative theta (RT) power spectra were observed during the exposure to *A. rugosa* essential oil when compared to that of no odor exposure. Whereas relative alpha (RA), relative slow alpha (RSA), spectral edge frequency 50% (SEF50), and spectral edge frequency 50% of alpha (ASEF) power spectra values significantly increased. These results reveal that the EEG power spectra changes incurred during the exposure to the essential oil of *A. rugosa* may be associated with the enhancement of freshness and concentration states of the human brain.

## 1. Introduction

In aromatherapy, essential oils from aromatic plants have been utilized to heal various psychophysiological disorders, including depression, anxiety, insomnia, and tension, and restoring physical, as well as emotional, conditions since the ancient era [1]. It is well known that the inhalation of essential oils can produce positive psychological and physiological functions in humans by reducing mental stress, and increasing mind relaxation and cognitive functions via stimulation of the central nervous system [2,3,4]. Previous studies have also proved that fragrances from essential oils can influence the mental condition of human beings [3,5,6,7,8,9,10]. In particular, the aromatic volatile components in essential oils, such as mono- and sesquiterpene hydrocarbons and their oxygenated derivatives, are mainly responsible for their characteristic fragrances. In day-to-day life, these fragrant molecules effectively influence the mood, stress, and working capacity of individuals [11].

The physiological changes stimulated through the fragrance inhalation of essential oils are highly associated with the regulation of the olfactory nervous system [12]. In the olfactory system, the olfactory mucosa finds fragrance molecules via inhalation of essential oils in the posterior and superior parts of the nasal cavity. The olfactory bulb receives the fragrance signal via the olfactory sensory neurons and supplies input to other brain regions that modify neuronal activity [13]. A number of electrophysiological techniques have been developed to examine brain function. Among them, the changes in neuronal activity due to the stimulation of fragrance inhalation can be easily measured by electroencephalography (EEG) [14,15]. EEG is an extensively used technique to determine the impact of fragrance on human brain functions. EEG is used to measure neuronal electrical activity in terms of brain waves. Brain waves are natural processes in the brain that appear during active, as well as resting, states. Our thoughts, emotions, and behavior mirror neuronal activity within the brain. EEG calculates these neuronal electrical activities and represents them as waves. The EEG spectra bands were categorized into five major waves: delta (0–4 Hz), theta (4–8 Hz), alpha (8–13 Hz) beta (13–30 Hz), and gamma (>30 Hz). Generally, the alterations in brain wave activities are highly interlinked with specific functions of the brain [13,16]. Previously, several studies have found that essential oils from a variety of plants, such as peppermint, lavender, rosemary, sandalwood, neroli, jasmine, bergamot, and *Abies* species, significantly altered EEG activity, resulting in the positive psychological and physiological functions of humans [4,11].

*Agastache rugosa* (Fisch. & C.A.Mey.) Kuntze (Lamiaceae) is a perennial herb and is widely distributed in East Asian Countries, including Korea, Japan, and China [17,18]. In Korea, *A. rugosa* is commonly called Korean mint (Baechohyang). The aerial parts of the plant have a unique scent, and the leaves are used as an ingredient for enhancing the aroma and taste of Korean dishes, especially in salads and soups [19,20,21]. *A. rugosa* has been traditionally used to cure various ailments, including anorexia, anxiety, bacterial infections, colds, cholera, diarrhea, dispel damp, fever, gas, halitosis, headaches, halitosis, nausea, miasma, and vomiting, etc. [18,19,21,22]. Previous studies indicated that the crude extracts and compounds of *A. rugosa* possess numerous therapeutic properties, such as antimicrobial [23], anti-inflammatory [24], cardiovascular [25,26], antioxidant [27], anti-atherogenic [28], anti-HIV [29], melanogenesis [30], anticancer [31], anti-photoaging [32], anti-adipogenic, and anti-lipogenic effects [33]. Furthermore, various phytochemical studies revealed the presence of methyl hexadecanoate, β-sitosterol, phenolic acid, ursolic acid, apigenin, protocatechuic acid [22], sterols, phenylpropanoids, flavonoids, lignans, and terpenoids [32] in *A. rugosa*. The essential oil obtained from the stem of *A. rugosa* contains various groups of aromatic components, such as alcohols, aldehydes, ketones, esters, and terpenoids [34]. In addition, rosmarinic acid, tilianin [35], and acacetin [36] are the main compounds in the extracts of *A. rugosa*. In particular, acacetin 7-O-β-d-glucoside (tilianin) attenuates house dust mite-induced allergic asthma in mice [37].

There are some reports on the essential oil composition of *A. rugosa* (Baechohyang) in South Korea [27,38]. The essential oil composition of plant species may vary depending on various ecological factors [39]. Although the essential oil of *A. rugosa* exhibits various biological activities, including antioxidant, antimicrobial, and antitumor potentials, etc. [26], the effect of exposure to *A. rugosa* essential oil on human EEG activity is still unknown. Therefore, the present study was initiated to determine the essential oil composition of Korean *A. rugosa* and to evaluate the effects of exposure to its essential oil on human EEG activity in order to utilize this essential oil in aromatherapy.

## 2. Materials and Methods

### 2.1. Plant Material and Cultivation

One hundred seeds of Korean domestic wild Baechohyang (*A. rugosa*) were collected in 2019–2020 and they were stored in a refrigerator before sowing. The seeds were sown in seedling trays (17 cm^3^, Seoul Bio, Seoul, Korea) filled with horticultural topsoil (rich farms) during April 2020. All seedlings in trays were grown until the plants reached the 3.5 leaf stage in the glass greenhouse of the Agricultural Research Institute, Gangwon-do Agricultural Research and Development Institute, where the proper temperature (23–25 °C day) and humidity were maintained. The seedlings were planted in a phosphorus field. After that, aerial parts of *A. rugosa* were harvested when it reached the flowering period and used for essential oil extraction.

### 2.2. Steam Distillation Extraction

The essential oil was extracted from the aerial parts of *A. rugosa* using the steam distillation extraction method. The steam distillation extraction was carried out with a 1 kg sample of *A. rugosa* for 90 min using a Clevenger-type apparatus. After extraction, water and impurities in the extracted essential oil were removed using anhydrous sodium sulfate. The yield (%) of the essential oil was calculated in triplicate as the volume (mL) of the extracted essential oil relative to the amount of the fresh plant sample (1 kg).

### 2.3. GC–MS Analysis of A. rugosa Essential Oil

The volatile aromatic components in the essential oil of *A. rugosa* were identified by gas chromatography and mass spectrometry (GC–MS) analysis. GC–MS analysis was carried out with a Varian CP-3800 (GC)/Varian 1200 L (MS) equipped with a VF-5MS polydimethylsiloxane capillary column (30 m × 0.25 mm × 0.25 μm). The GC oven temperature was kept at 50 °C for 5 min, then heated to 250 °C at a rate of 3 °C/min, and maintained for 15 min. One μL of the sample was injected with a split ratio of 10:1, and helium was used as a carrier gas at the rate of 1 mL/min. The injector temperature was set at 250 °C and the ion source temperature was set at 200 °C. For MS analysis, the ionization voltage was set to 70 eV, and the mass range was set to 50–500 m/z. The components in the essential oil of *A. rugosa* were identified by comparing the mass spectrum data of the NIST library and the retention indices (RI) relative to a homologous series of n-alkanes (C_8_–C_20_) with those reported in the literature [40].

### 2.4. Odor Evaluation

A sensory evaluation was performed to determine the fragrance of *A. rugosa* essential oil. For sensory evaluation, 1% of dilute *A. rugosa* essential oil was prepared using a colorless and odorless dipropylene glycol (DPG) solvent, and it was evaluated by three expert panels with olfactory training. The diluted essential oil was placed on the lower part of the commercial odor paper strip. Then the odor characteristics of the essential oil were recorded according to the odor type felt by the professional panel.

### 2.5. EEG Study

The study followed the Declaration of Helsinki on Biomedical Research Involving Human Subjects and was approved by the ethics committee from the Kangwon National University (KWNUIRB-2021-11-007-002), Chuncheon, Korea.

#### 2.5.1. Subjects

Thirty-eight right-handed healthy volunteers (19 men and 19 women) aged between 20 and 30 years participated in this study. The mean ages of men and women were 23.5 ± 3.2 and 22.8 ± 2.7, respectively. The inclusion criteria for the subjects were non-smokers and right-handed without any abnormalities in olfaction. None of the subjects had olfactory diseases or abused drugs. Alcohol consumption or medications were prohibited from 2 days before the experiment. There were no statistically significant differences between men and women. All the subjects were students and no one refused to participate in this study. All subjects gave their informed consent before participation in the EEG study.

#### 2.5.2. Experimental Design

In this study, a single group pre-test and post-test experimental design was used for 38 subjects. A careful measurement was carried out before and during the exposure to the essential oil. The subjects were informed that the aim of this study was to evaluate the changes of EEG activity during no odor and essential oil exposure. The subjects were instructed to sit quietly, close their eyes, and breathe normally during the EEG measurement. After the measurement, the subjects were requested to provide their preference and impression of the tested essential oil.

#### 2.5.3. EEG Recordings

**Figure 1:**
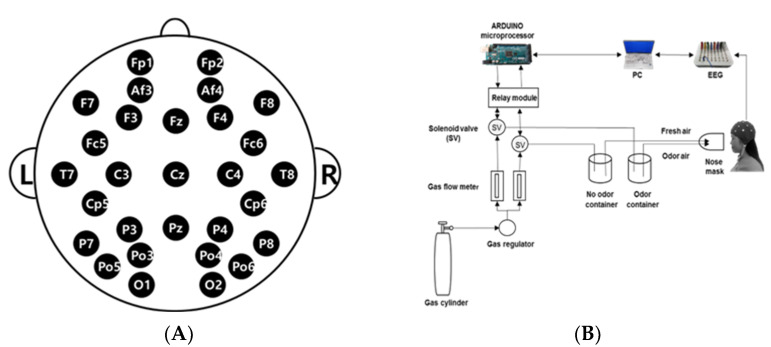
EEG experiment: (**A**) EEG electrode placement locations using the International 10–20 system; (**B**) Schematic diagram of the experimental procedure.

For EEG measurement, a Quick-30 Dry EEG Headset (Cognionics Inc., San Diego, CA, USA) was used, and EEG data were recorded with Cognionics Data Acquisition Software (Cognionics Inc., USA). The EEG recordings were made using an electrode cap from 29 channels positioned on the scalp at Fp1, Fp2, Af3, Af4, F3, F4, Fz, F7, F8, Fc5, Fc6, C3, C4, T7, T8, Cp5, Cp6, P3, P4, P7, P8, Cz, Pz, O1, O2, Po3, Po4, Po5, and Po6 regions according to the International 10–20 System (Figure 1A). The electrodes were referenced to the ipsilateral earlobe electrodes. The EEG sampling rate of the measured subjects was 500 Hz, filtered in the range of 4–50 Hz, and the readings were stored in a computer by 24-bit analog-to-digital conversion. The electrodes (silver/silver chloride) were applied over an elastic cap with plastic electrode holders. The ECI electrode gel (Electro-gel™, Electro-Cap International Inc., Eaton, OH, USA) was applied to each electrode to connect with the surface of the scalp to drop the electric resistance of the scalp below 5 kΩ [41].

#### 2.5.4. Fragrance Administration

The essential oil of *A. rugosa* was used as the fragrance stimulus. The stimulus was presented to the subjects in a randomized sequence. The EEG recording room was maintained with a constant temperature (25 °C) and humidity (50%). The diluted *A. rugosa* essential oil (1%) was placed in the sample container and an EEG measurement was performed for a total of 120 s with 60 s of air with no odor and 60 s of air with *A. rugosa* essential oil. The odorless fresh air was pumped into the chamber at the rate of 3 L/min. The air outflow chamber was placed 5 cm in front of the subject’s nose (Figure 1B).

#### 2.5.5. Data Analysis

The EEG power spectrum values [microvolt square (𝜇V2)] were calculated for 25 EEG analysis indicators (Table 1). To remove noise, a 1–1000 Hz band pass filter was employed with a 24 dB/octave roll-off, and a 60 Hz notch filter was applied. The t-mapping of EEG waves was constructed using the Telescan software package (LAXTHA Inc., Daejeon, Korea). Out of 60 s EEG data recorded, only 50 s EEG data were analyzed for each condition, such as air with no odor and air with *A. rugosa* essential oil. The SPSS statistical package 26 (IBM Inc.) was used to determine significant differences in EEG activity between air with no odor and air with *A. rugosa* essential oil, using a paired Student’s *t*-test. The *p* value < 0.05 was considered significant.

## 3. Results

### 3.1. Chemical Composition of the Essential Oil from the Aerial Parts of A. Rugosa

The essential oil extracted from the aerial parts of *A. rugosa* was transparent lemon in color and aromatic, herbal, oily, and spicy. The yield of steam distilled *A. rugosa* essential oil was 0.15 ± 0.02% (*v/w*). In the essential oil of *A. rugosa*, a total of 29 volatile components were identified based on the retention indices and mass spectra data, which accounted for 99.72 ± 0.13 of the total oil. The identified components are listed in order of their elution from a VF-5MS column. The essential oil of *A. rugosa* contains 10 sesquiterpenes, 9 monoterpenes, 3 phenylpropanoids, 2 esters, 1 alcohol, 1 ketone, 1 aldehyde, 1 hydrocarbon, and 1 phenol. The most abundant component in the essential oil of *A. rugosa* essential was estragole (89.49%), followed by D-limonene (3.40%), menthone (1.80%), and pulegone (1.86%). The concertation of the remaining components in the essential oil was less than 1%.

### 3.2. Effect of A. Rugosa Essential Oil on Human EEG Study

In this study, the essential oil of *A. rugosa* was used to stimulate the olfactory system. EEG power spectrum values were measured during the air with no odor and air with *A. rugosa* essential oil odor. The changes of 25 EEG indices were analyzed from 29 electrodes located on the scalp. As a result of EEG measurement in both genders, there were significant differences in six indices among the 25 EEG indices analyzed. Absolute theta (AT) and relative theta (RT) power spectra significantly decreased at different sites during exposure to *A. rugosa* essential oil. On the other hand, significant increases in relative alpha (RA) and relative slow alpha (RSA) spectral edge frequency 50% (SEF50), and spectral edge frequency 50% of alpha (ASEF) values were observed due to *A. rugosa* essential oil exposure when compared with no odor exposure (Table 2).

**Figure 2:**
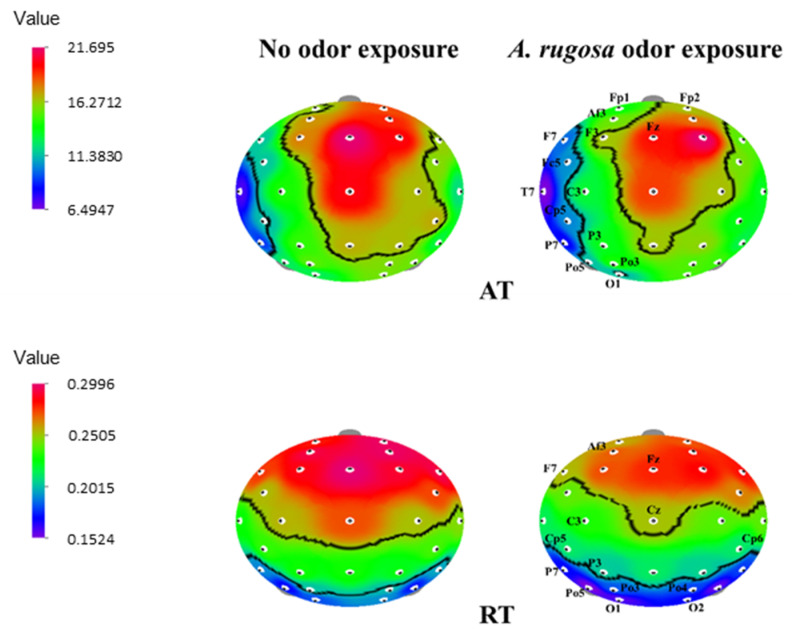
The t-mapping of EEG power spectrum changes during no odor and *A. rugosa* essential oil odor conditions. AT, absolute theta; RT, relative beta. The marked sites in the t-mapping denote the significant changes during exposure to *A. rugosa* essential oil.

During exposure to *A. rugosa* essential oil, AT values significantly decreased in the frontal region (Fp1, Fp2, Af3, F7, F3, Fz, and Fc5), temporal region (T7, C3, and Cp5), parietal region (P7 and P3), and occipital region (Po5, Po3, and O1) (*p* < 0.05). In the case of RT spectrum, a significant decrease was observed in the frontal region (Af3, F7, and Fz), temporal region (C3, Cz, Cp5, and Cp6), parietal region (P7 and P3), and occipital region (Po5, Po3, Po4, O1, and O2) (Table 3 and Figure 2).

**Figure 3:**
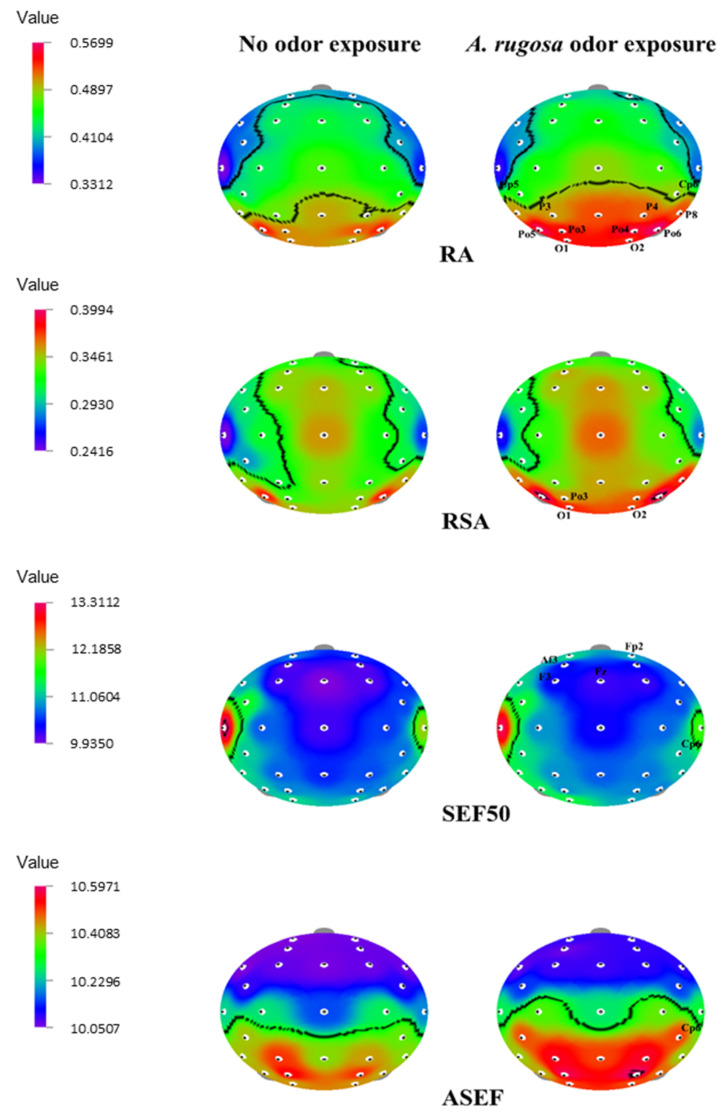
The t-mapping of EEG power spectrum changes during no odor and *A. rugosa* essential oil odor conditions. ASEF, spectral edge frequency 50% of alpha; RA, relative alpha; RSA, relative slow alpha; SEF50, spectral edge frequency 50%. The marked sites in the t-mapping denote the significant changes during exposure to *A. rugosa* essential oil.

On the other hand, the RA power spectrum significantly increased (*p* < 0.05) in the temporal region (Cp5, and Cp6), parietal region (P8, P3, and P4), and overall occipital region (Po5, Po6, Po3, Po4, O1, and O2). Slow alpha waves increased significantly in the occipital lobe (Po3, O1, and O2) (*p* < 0.05). Furthermore, SEF50 values showed that the left frontal regions (Fp2 and Af3), frontal regions (F3 and Fz), and temporal region (Cp6) significantly increased. The ASEF index significantly increased at the right temporal region, Cp6 (*p* < 0.05) (Table 4 and Figure 3).

## 4. Discussion

Plants of the Lamiaceae family are extensively utilized for essential oils. Monoterpenes and sesquiterpenes are important essential oil constituents in a variety of aromatic plants. Among them, *A. rugosa* is an important traditional medicinal plant in Korea. In total, 29 chemical compounds have been identified in Korean-grown *A. rugosa* essential oil by GC–MS analysis. In the present study, estragole (89.49%) was the most abundant component in the essential oil, followed by D-limonene (3.40%), menthone (1.80%), and pulegone (1.86%). Similar to our findings, Lim et al. [38] found that estragole (84.25%) was the predominant compound in the essential oil of *A. rugosa* leaves. Yamani et al. [50] also reported that estragole was the main component in the essential oil of Australian-grown *A. ruogsa.* The major component estragole has analgesic properties [12]. In the essential oil from the leaves of *A. rugosa* collected in China, p-menthan-3-one (48.8%) was the main component, followed by estragole (20.8%) [31]. Another study demonstrated that methyl eugenol (50.51%), estragole (8.55%), eugenol (7.54%), thymol (3.62%), pulegone (2.56%), limonene (2.49%), and caryophyllene (2.38%) were major components in the essential oil of *A. rugosa* [51]. p-Menthan-3-one (48.8%), estragole (20.8%), monoterpenes (8.3%), and oxygenated terpenes (5.6%) were identified in *A. rugosa* leaf [52]. The variations in the essential oil yield and its composition could be influenced by various factors, including the cultivation techniques, geographical location, age of the plant, and climatic conditions, etc. [53].

Recently, several studies have been conducted on the psychophysiological properties of essential oil components using animal models. However, only a few studies have been investigated to determine the potential effectiveness of essential oils in humans [54]. Previous studies showed that aromatic components exhibit a positive change, such as improving alertness and concentration, increasing relaxation, and attenuating mental stress and tension via stimulation of the central nervous system [7,8]. EEG is widely used to evaluate the neurophysiological function of the human brain. With this background, we attempted to evaluate whether exposure to *A. rugosa* essential oil exhibits any effect on human EEG activity. In this study, changes in the 25 EEG power spectrum indices between air with no odor and air with *A. rugosa* essential oil odor were analyzed. During inhalation of *A. rugosa* essential oil, AT (4–8 Hz) and RT (4–8/4–50 Hz) activities were significantly decreased in different sites, whereas RA (8–13/4–50 Hz), RSA (8–11/4–50 Hz), SEF50 (4–50 Hz), and ASEF (8–13 Hz) were increased in different sites.

In the study, the AT value was significantly decreased in different brain regions, such as the frontal, temporal, parietal, and occipital regions during exposure to *A. rugosa* essential oil. Sowndhararajan et al. [9] also reported that absolute theta wave activity significantly decreased at the sites of FP1, FP2, F3, F4, T4, P3, and P4 during inhalation of the essential oil of *Inula helenium* root. In another study, AT wave activity significantly varied via left and right inhalation of aldehyde C10 odor when compared with inhalation via both nostrils [10]. During inhalation of the essential oil of *A. koreana* twigs, AT wave activity also significantly changed in the F3 and P4 regions [7]. The inhalation of *Abies sibirica* essential oil effectively reduced arousal levels by increasing theta waves [55]. The brain regions (lones) are classified into five major regions: frontal, temporal, parietal, and occipital regions, and each lobe is associated with different functions. Theta waves mainly appear during deep meditation and are also found in hippocampal and cortical regions. Theta waves are linked with the subconscious mind that controlles sleep, drowsiness, imaginative thinking, and creativity [16,56]. The reduction in theta wave activity is interrelated with memory formation. Further, during the implementation of a difficult task, theta waves have been believed to maintain attention [57]. The significant changes in AT due to the inhalation of *A. rugosa* essential oil may be associated with the drowsy or meditative state of the brain.

In the case of the RT power spectrum, significant decreases were noticed in frontal, temporal, parietal, and occipital regions during exposure to *A. rugosa* essential oil. RT wave activity also decreased at the site during inhalation of *Inula helenium* root essential oil (at FP1, FP2, F3, and F4 regions) and essential oil from the twigs of *A. koreana* (at F4 and P4 regions) [7,9]. Kim et al. [8] reported that the RT spectrum markedly decreased in the FP1 and P4 regions during inhalation of black pepper essential oil.

In the present study, RA, RSA, SEF50, and ASEF significantly increased in different brain regions during exposure to *A. rugosa* essential oil. Previous studies reported that the ASEF index significantly decreased during inhalation of *A. koreana* and black pepper essential oils [7,8]. However, ASEF spectrum activity increased in the Cp6 region due to *A. rugosa* essential oil exposure. Similarly, ASEF activity increased in the FC1, T8, AF4, and FPZ regions due to geosmin exposure [6]. Significant increases in the RA and RSA indices were observed during inhalation of essential oil of *A. koreana* twigs [7]. The SEF50 spectrum significantly decreased during inhalation of geosmin odor, black pepper essential oil, and supercritical carbon dioxide extract from the root of *A. gigas* [6,8,58]. The SEF50 is defined as the frequency below 50% of the overall EEG power and it specifies the spectral features of EEG data. Some reports indicated that the spectral edge frequency increased during light anesthesia conditions [49]. The significant changes of SEF50 due to *A. rugosa* essential oil inhalation may increase the concentration state of the brain function. In addition, previous studies found that gender and nostril variations play a major role in the EEG activity of different fragrances [7,8]. Another study indicated that men and women responded inversely during the exposure to fragrances [59]. Furthermore, gender variation occurred in the EEG activity of resting, stimulus, and non-stimulus conditions [8].

The data of this study clearly demonstrate that *A. rugosa* essential oil effectively stimulates brain wave activity in different regions of the brain. Furthermore, the major component, estragole, in the essential oil of *A. rugosa* may play a key role in the odor characteristics of this essential oil, thereby producing significant changes in EEG activity. Although exposure to the essential oil of *A. rugosa* produces significant changes in EEG activity, further studies are required in connection with different concentrations of an odor stimulus, and slightly longer EEG recording time with a placebo control and other commercial odor controls.

## 5. Conclusions

The GC–MS analysis revealed that the essential oil of *A. rugosa* mainly contains estragole. In the EEG study, exposure to *A. rugosa* essential oil exhibited significant decreases in absolute theta and relative theta power spectra and increases in relative alpha, relative slow alpha, spectral edge frequency 50%, and spectral edge frequency 50% of alpha spectra values. These EEG changes suggest that exposure to *A. rugosa* essential oil may be associated with the enhancement of the freshness and concentration states of the human brain. This essential oil can be used in aromatherapy for positive psychophysiological conditions.

## Figures and Tables

**Table 1:** The abbreviations, full names, and wavelength ranges of the EEG power spectrum indices.

No.	Indices	EEG Power Spectrum Indicators	Wavelength Range (Hz)	Condition
1	AT	Absolute theta power spectrum	4–8	Drowsiness [42]
2	AA	Absolute alpha power spectrum	8–13	Relaxation [43]
3	AB	Absolute beta power spectrum	13–30	Concentration or Alertness [44]
4	AG	Absolute gamma power spectrum	30–50	High level of cognition [45]
5	ASA	Absolute slow alpha power spectrum	8–11	Relaxation
6	AFA	Absolute fast alpha power spectrum	11–13	Creative or focused [43]
7	ALB	Absolute low beta power spectrum	12–15	Attention or alert [46]
8	AMB	Absolute mid beta power spectrum	15–20	Concentration or attention [46]
9	AHB	Absolute high beta power spectrum	20–30	Stress or tension [46]
10	RT	Relative theta power spectrum	4–8/4–50	
11	RA	Relative alpha power spectrum	8–13/4–50	
12	RB	Relative beta power spectrum	13–30/4–50	
13	RG	Relative gamma power spectrum	30–50/4–50	
14	RFA	Relative fast alpha power spectrum	11–13/4–50	
15	RSA	Relative slow alpha power spectrum	8–11/4–50	
16	RLB	Relative low beta power spectrum	12–15/4–50	
17	RMB	Relative mid beta power spectrum	15–20/4–50	
18	RHB	Relative high beta power spectrum	20–30/4–50	
19	RST	Ratio of SMR to theta	12–15/4–8	Unfocused attention ~ vigilance [47]
20	RMT	Ratio of mid beta to theta	15–20/4–8	Focused attention ~ concentration [48]
21	RSMT	Ratio of (SMR ~ mid beta) to theta	12–20/4–8	Attention
22	RAHB	Ratio of alpha to high beta	8–13/20–30	Relaxation index
23	SEF50	Spectral edge frequency 50%	4–50	Activity index [49]
24	SEF90	Spectral edge frequency 90%	4–50	Stress index [49]
25	ASEF	Spectral edge frequency 50% of alpha spectrum band	8–13	Refreshment

**Table 2:** Overall significant decreases and increases in EEG activity during exposure to *A. rugosa* essential oil.

No.	Indices	Variation	Sites	Condition
1	AT	Decreased (↓)	Fp1, Fp2, Af3, F7, F3, Fz, Fc5, T7, C3, Cp5, P3, P7, Po5, Po3, O1	Drowsiness or meditation
2	RT		Af3, F7, Fz, C3, Cz, Cp5, Cp6, P7, P3, Po5, Po3, Po4, O1, O2	
3	RA	Increased (↑)	Cp5, Cp6, P8, P3, P4, Po5, Po6, Po3, Po4, O1, O2	Stabilized brain
4	RSA		Po3, O1, O2	Ready to concentrate
5	SEF50		Fp2, Af3, F3, Fz, Cp6	Activity index
6	ASEF		Cp6	Refreshment index

AT, absolute theta; RT, relative alpha; RA, relative alpha; RSA, relative slow alpha; SEF50, spectral edge frequency 50%; ASEF, spectral edge frequency 50% of alpha. The *p* value < 0.05 was considered significant.

**Table 3:** Significant changes of AT and RT power spectra between no odor exposure and *A. rugosa* essential oil odor exposure.

Indices	Site	No Odor (μV^2^)	*A. rugosa* Odor (μV^2^)	*t* Test	*p* Value *
AT	Fp1	16.071 ± 13.698	14.014 ± 11.474	2.428	0.020
	Fp2	18.459 ± 14.207	16.477 ± 13.358	2.116	0.041
	Af3	17.304 ± 13.665	15.502 ± 12.132	3.105	0.004
	F7	11.556 ± 7.468	9.902 ± 7.125	3.267	0.002
	F3	17.589 ± 12.825	16.424 ± 11.760	2.735	0.010
	Fz	21.160 ± 16.226	19.328 ± 14.260	2.687	0.011
	Fc5	11.551 ± 7.763	10.477 ± 7.476	3.072	0.004
	T7	7.296 ± 5.832	6.495 ± 5.051	2.789	0.008
	C3	14.259 ± 9.798	13.207 ± 9.649	3.032	0.004
	Cp5	11.325 ± 7.254	10.383 ± 7.036	2.543	0.015
	P7	9.158 ± 5.993	8.221 ± 6.304	2.996	0.005
	P3	14.635 ± 9.198	13.589 ± 9.564	3.231	0.003
	Po5	12.671 ± 7.783	11.444 ± 8.011	2.640	0.012
	Po3	15.224 ± 9.630	14.300 ± 9.697	2.327	0.026
	O1	12.706 ± 7.474	11.289 ± 7.177	2.856	0.007
RT	Af3	0.293 ± 0.119	0.270 ± 0.105	2.362	0.024
	F7	0.275 ± 0.099	0.254 ± 0.101	2.174	0.036
	Fz	0.300 ± 0.119	0.278 ± 0.105	2.398	0.022
	C3	0.255 ± 0.094	0.231 ± 0.083	2.959	0.005
	Cz	0.272 ± 0.103	0.253 ± 0.094	2.242	0.031
	Cp5	0.234 ± 0.086	0.213 ± 0.080	2.791	0.008
	Cp6	0.237 ± 0.096	0.213 ± 0.084	2.140	0.039
	P7	0.191 ± 0.090	0.171 ± 0.087	2.587	0.014
	P3	0.229 ± 0.094	0.205 ± 0.089	2.863	0.007
	Po5	0.170 ± 0.100	0.152 ± 0.095	2.400	0.022
	Po3	0.202 ± 0.102	0.184 ± 0.184	2.085	0.044
	Po4	0.200 ± 0.107	0.181 ± 0.099	2.087	0.044
	O1	0.183 ± 0.102	0.159 ± 0.093	2.712	0.010
	O2	0.193 ± 0.110	0.171 ± 0.097	2.141	0.039

AT, absolute theta; RT, relative theta. * The *p* value < 0.05 was considered significant; number of participants = 38 (19 men and 19 women).

**Table 4:** Significant changes of RA, RSA, SEF50 and ASEF power spectra between no odor exposure and *A. rugosa* essential oil odor exposure.

Indices	Site	No Odor (μV^2^)	*A. rugosa* Odor (μV^2^)	*t* Test	*p* Value *
RA	Cp5	0.426 ± 0.136	0.453 ± 0.133	−2.521	0.016
	Cp6	0.454 ± 0.134	0.486 ± 0.133	−2.330	0.025
	P8	0.492 ± 0.146	0.523 ± 0.149	−2.419	0.021
	P3	0.474 ± 0.145	0.505 ± 0.147	−2.239	0.031
	P4	0.489 ± 0.142	0.520 ± 0.142	−2.226	0.032
	Po5	0.536 ± 0.168	0.561 ± 0.179	−2.043	0.048
	Po6	0.536 ± 0.155	0.569 ± 0.153	−2.410	0.021
	Po3	0.509 ± 0.156	0.541 ± 0.167	−2.129	0.040
	Po4	0.522 ± 0.151	0.555 ± 0.161	−2.156	0.038
	O1	0.508 ± 0.163	0.539 ± 0.179	−2.412	0.021
	O2	0.498 ± 0.165	0.535 ± 0.165	−2.556	0.015
RSA	Po3	0.328 ± 0.173	0.356 ± 0.174	−2.185	0.035
	O1	0.349 ± 0.186	0.375 ± 0.192	−2.099	0.043
	O2	0.343 ± 0.190	0.370 ± 0.183	−2.050	0.047
SEF50	Fp2	10.521 ± 2.376	10.976 ± 2.740	−2.333	0.025
	Af3	10.076 ± 1.109	10.362 ± 1.190	−2.833	0.007
	F3	10.141 ± 1.014	10.329 ± 1.103	−2.406	0.021
	Fz	9.935 ± 0.996	10.171 ± 1.030	−2.457	0.019
	Cp6	10.747 ± 1.678	11.063 ± 1.871	−2.234	0.032
ASEF	Cp6	10.376 ± 0.630	10.492 ± 0.553	−2.178	0.036

RA, relative alpha; RSA, relative slow alpha; SEF50, spectral edge frequency 50%; ASEF, spectral edge frequency 50% of alpha. * The *p* value < 0.05 was considered significant; number of participants = 38 (19 men and 19 women).

## Data Availability

The data presented in this study are available within the article.
